# Supervised pulmonary hypertension exercise rehabilitation (SPHERe): study protocol for a multi-centre randomised controlled trial

**DOI:** 10.1186/s12890-020-01182-y

**Published:** 2020-05-19

**Authors:** Gordon McGregor, Julie Bruce, Stuart Ennis, James Mason, Ranjit Lall, Chen Ji, Harbinder Sandhu, Kate Seers, Prithwish Banerjee, Alastair Canaway, Katie Booth, Stephanie J. C. Taylor, Elizabeth Robertson, Tamar Pincus, Sally Singh, David Fitzmaurice, Sarah Bowater, Paul Clift, Martin Underwood

**Affiliations:** 1 Department of Cardiopulmonary Rehabilitation, Centre for Exercise & Health, University Hospitals Coventry & Warwickshire NHS Trust, Coventry, UK; 2 Warwick Clinical Trials Unit, Warwick Medical School, University of Warwick, Coventry, UK; 3 Centre for Sport Exercise & Life Sciences, Coventry University, Coventry, UK; 4 Centre for Health Economics at Warwick, Warwick Medical School, University of Warwick, Coventry, UK; 5 Warwick Research in Nursing, Warwick Medical School, University of Warwick, Coventry, UK; 6 Department of Cardiology, University Hospitals Coventry & Warwickshire NHS Trust, Coventry, UK; 7 Institute of Population Health Sciences, Barts and The London School of Medicine and Dentistry, Queen Mary University of London, London, UK; 8 Patient & Public Involvement Representative, Leicester, UK; 9 Department of Psychology, Royal Holloway University of London, London, UK; 10 Centre for Exercise and Rehabilitation Science, University Hospitals of Leicester NHS Trust, Leicester, UK; 11 Department of Cardiology, University Hospitals Birmingham NHS Foundation Trust, Birmingham, UK

**Keywords:** Pulmonary hypertension, Cardiac rehabilitation, Pulmonary rehabilitation, Randomised controlled trial, Complex intervention

## Abstract

**Background:**

Supervised cardio-pulmonary rehabilitation may be safe and beneficial for people with pulmonary hypertension (PH) in groups 1 (pulmonary arterial hypertension) and 4 (chronic thromboembolic disease), particularly as a hospital in-patient. It has not been tested in the most common PH groups; 2 (left heart disease), 3 (lung disease), or 5 (other disorders). Further it has not been evaluated in the UK National Health Service (NHS) out-patient setting, or with long-term follow-up. The aim of this randomised controlled trial (RCT) is to test the clinical and cost-effectiveness of a supervised exercise rehabilitation intervention with psychosocial support compared to best practice usual care for people with PH in the community/outpatient setting.

**Methods:**

This multi-centre, pragmatic, two-arm RCT with embedded process evaluation aims to recruit 352 clinically stable adults with PH (groups 1–5) and WHO functional class II-IV. Participants will be randomised to either the Supervised Pulmonary Hypertension Exercise Rehabilitation (SPHERe) intervention or control. The SPHERe intervention consists of 1) individual assessment and familiarisation sessions; 2) 8-week, twice-weekly, supervised out-patient exercise training; 3) psychosocial/motivational support and education; 4) guided home exercise plan. The control intervention consists of best practice usual care with a single one-to-one practitioner appointment, and general advice on physical activity. Outcomes will be measured at baseline, 4 months (post-intervention) and 12 months by researchers blinded to treatment allocation. The primary outcome is the incremental shuttle walk test at 4 months. Secondary outcomes include health-related quality of life (HRQoL), time to clinical worsening and health and social care use. A purposive sample of participants (*n* = 20 intervention and *n* = 20 control) and practitioners (*n* = 20) will be interviewed to explore experiences of the trial, outcomes and interventions.

**Discussion:**

The SPHERe study is the first multi-centre clinical RCT to assess the clinical and cost effectiveness of a supervised exercise rehabilitation intervention compared to usual care, delivered in the UK NHS, for people in all PH groups. Results will inform clinicians and commissioners as to whether or not supervised exercise rehabilitation is effective and should be routinely provided for people with PH.

**Trial registration:**

ISRCTN no. 10608766, prospectively registered on 18th March 2019.

## Background

Pulmonary hypertension (PH) is a debilitating condition causing dyspnoea, fatigue, palpitations, dizziness, and chest pain [1]. Many affected people are anxious about, and avoid, physical activity. Depression is common, and quality of life (QoL) can be poor [2]. There are five diagnostic groups: Group 1 pulmonary arterial hypertension (PAH); Group 2 PH due to left heart disease; Group 3 PH due to lung diseases or hypoxia; Group 4 chronic thromboembolic PH (CTEPH); Group 5 PH due to other disorders [3]. Drug treatment and pulmonary endarterectomy may help people with PAH [4] and CTEPH [5], respectively, but benefit is often limited. For people with PH secondary to cardiac or pulmonary disease (groups 2 & 3), there are no treatments of proven benefit [6, 7].

Exercise rehabilitation appears to be safe and may help people with PAH and CTEPH, particularly when undertaken as a hospital in-patient [8]. Recent recommendations support a conservative approach, under the careful supervision of appropriately skilled practitioners [3, 9]. However, exercise rehabilitation has not been tested in those with the most common forms of PH (groups 2 & 3), or in an out-patient setting in the UK [6, 7, 10]. For people living with chronic heart failure (CHF) or chronic obstructive pulmonary disease (COPD), without co-existing PH, exercise rehabilitation is recommended by NICE [11], and the British Thoracic Society [12], supported by a considerable evidence base [13]. Exercise rehabilitation can improve fitness in these populations, and increase ability to ‘self-manage’, often reducing health and care utilisation [13, 14]. Thus, it is plausible, that exercise may also help people in other PH groups with underlying cardiac and pulmonary disease [7, 15].

A 2017 Cochrane review of exercise rehabilitation for PH identified six RCTs (N = 206 mainly people with PAH or CTEPH) with short follow-up (3–15 weeks) [16]. Low quality evidence showed that exercise rehabilitation programmes increased six-minute walk test (6MWT) distance by 60 m, compared to usual care (95% CI 30 m to 90 m), without any serious adverse events [17]. The SF-36 physical component score improved by 4.63 points (95% CI 0.80 to 8.47), but this was not considered clinically important. Few studies have tested exercise rehabilitation for PH secondary to cardiac and pulmonary disease (groups 2 & 3) [18, 19].

### Rationale for a trial

In-patient exercise rehabilitation may have short-term benefit on exercise capacity in selected people with PAH or CTEPH. However, it is not known if these benefits extend to people in PH groups 2, 3, & 5, if exercise rehabilitation delivered in an NHS out-patient setting is effective, or if there are any long-term health benefits or harms. To date there have been no high-quality, multi-centre RCTs to test the clinical and cost effectiveness of supervised exercise rehabilitation with psychosocial support compared to best-practice usual care delivered in the UK NHS out-patient setting.

## Methods/design

### Aims and objectives

The aim of the trial is to assess the clinical and cost-effectiveness of the Supervised Pulmonary Hypertension Exercise Rehabilitation (SPHERe) intervention compared to best-practice usual care for people with PH.

### Objectives

The objective is to run a definitive multi-centre pragmatic RCT testing the clinical and cost-effectiveness of the SPHERe intervention compared to best-practice usual care, including:

1. A pre-pilot to test feasibility, refine intervention delivery and manualised practitioner training, and prepare trial set-up at selected centres;
2. An internal pilot, with formative process evaluation, at a sample of out-patient centres to test recruitment and trial procedures;
3. A main trial with embedded process evaluation.

### Trial design and setting

**Fig. 1:**
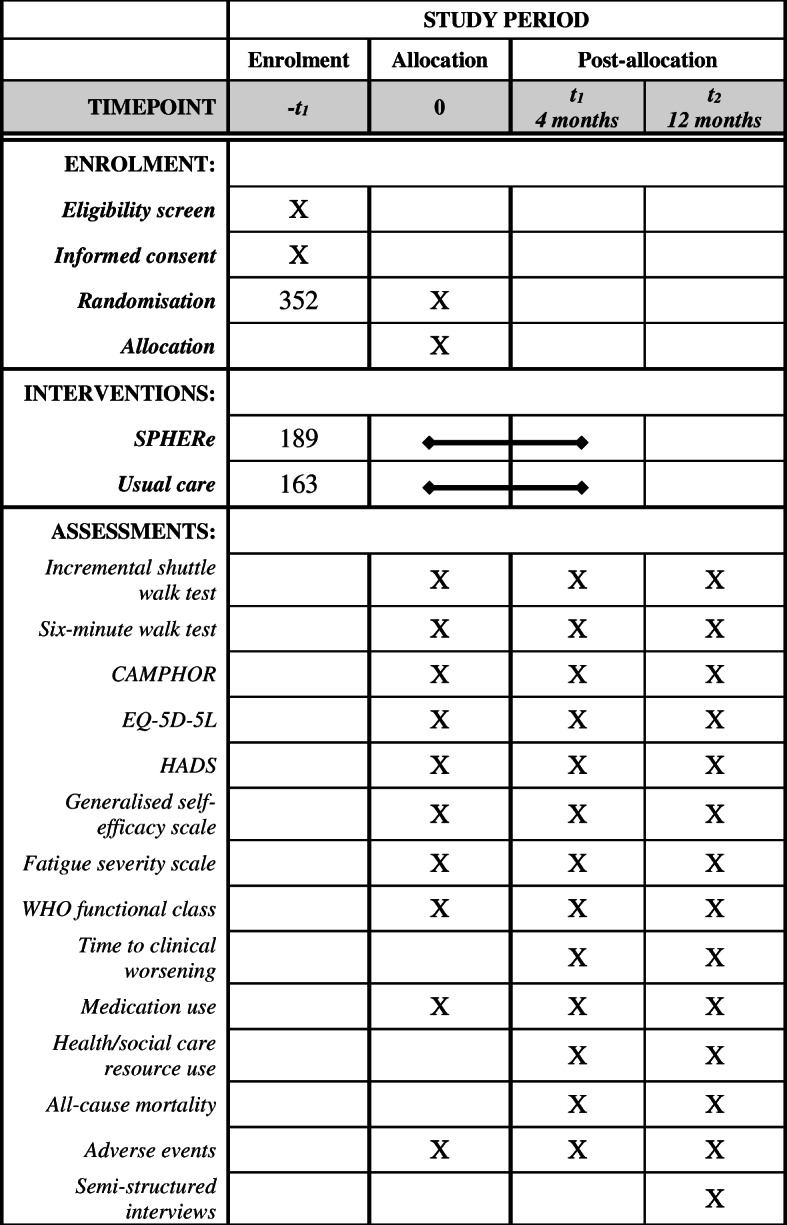
Schedule of enrolment, interventions, and assessments

**Fig. 2:**
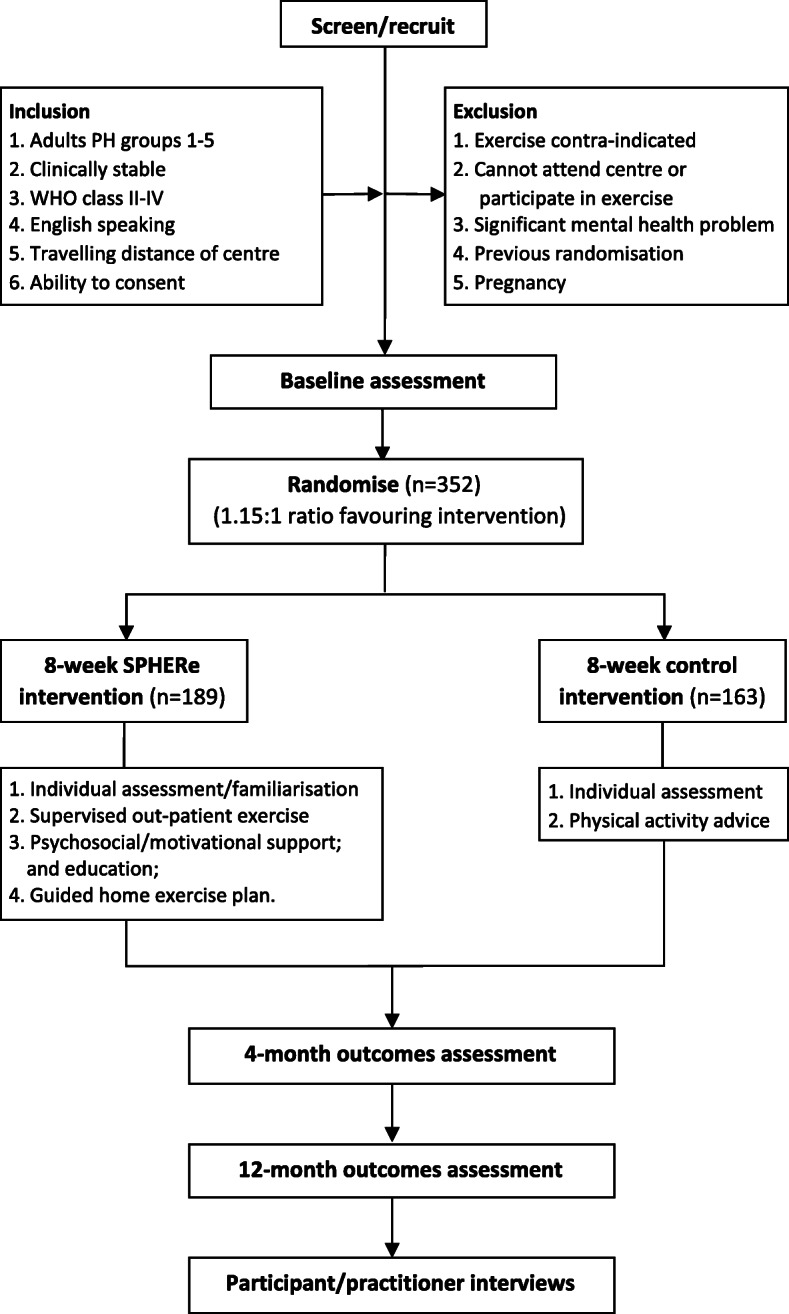
Trial flow chart

This protocol follows guidance from the Standard Protocol Items: Recommendations for Interventional Trials (SPIRIT) [20]. A SPIRIT schedule of enrolment, interventions and assessment is provided in Fig. 1 and a SPIRIT checklist is provided in Additional File 1. SPHERe is a multicentre, pragmatic, parallel, two-arm RCT with internal pilot study and embedded economic evaluation and qualitative study **(**Fig. 2**).** The trial will recruit from up to 20 NHS cardio-pulmonary rehabilitation centres in England. Participants will be randomised in a 1.15:1 ratio between intervention and control arms. Trial methods and design are summarised in the World Health Organization (WHO) Trial Registration Data Set **(**Table 1**).**

**Table 1:** World Health Organization Trial Registration Data Set

Data category	Information
Primary registry and trial identifying number	ISRCTN10608766
Date of registration in primary registry	18th March 2019
Secondary identifying numbers	REC reference: 19/WM/0155 NIHR HTA reference: 17/129/02
Source(s) of monetary or material support	NIHR Health Technology Assessment grant
Primary sponsor	UHCW NHS Trust University Hospital Clifford Bridge Rd., Coventry CV2 2DX Tel: 02476 966,195 Email: R&DSponsorship@uhcw.nhs.uk
Secondary sponsor(s)	n/a
Contact for public queries	SPHERe Resource Warwick Clinical Trials Unit Tel: 02476150285 Email: sphere@warwick.ac.uk
Contact for scientific queries	Dr Gordon McGregor Warwick Clinical Trials Unit Tel: 02476150285 Email: gordon.mcgregor@warwick.ac.uk
Public title	Supervise exercise rehabilitation for people with pulmonary hypertension
Scientific title	Supervised Pulmonary Hypertension Exercise REhabilitation (SPHERe): a multi-centre randomised controlled trial
Countries of recruitment	England
Health condition(s) or problem(s) studied	Pulmonary hypertension (PH) (groups 1–5)
Intervention(s)	Intervention group: 1) Individual assessment and exercise familiarisation, 2) Supervised out-patient exercise programme, 3) Psychosocial and motivational support; and education, 4) Guided home exercise plan. Control intervention: Best practice usual care
Key inclusion and exclusion criteria	Inclusion: Adults (18+) with confirmed PH (groups 1 to 5), clinically stable, WHO functional class II, III or IV, fluent in spoken English, travelling distance of a SPHERe centre, ability to provide informed consent. Exclusion: Absolute contra-indications to exercise, PH-related complications, or comorbidities severe enough to prevent attendance at a SPHERe centre, or exercise, mental health issue preventing engagement with trial procedures, previous randomisation in SPHERe, pregnant at time of recruitment
Study type	Type: Pragmatic, interventional, multi-centre Allocation: randomised Assignment: parallel Masking: outcomes assessors, chief investigator, statistician
Date of first enrolment	15th January 2020
Target sample size	382
Recruitment status	Recruiting
Primary outcome(s)	Incremental shuttle walk test at four months
Key secondary outcomes	At 4 and 12 months: Six-minute walk test, Cambridge Pulmonary Hypertension Outcome Review, Hospital Anxiety and Depression Scale, Generalised self-efficacy scale, Fatigue Severity Scale, WHO functional class, Medication use, Time to clinical worsening, Hospital admissions, Adverse events, All-cause mortality, EQ-5D-5 L, Health and care resource use. At 12 months: Semi-structured interviews with participants and practitioners

Table 1 World Health Organization (WHO) Trial Registration Data Set.

### Pre-pilot feasibility study

A pre-pilot feasibility phase was undertaken (August – December 2019) to complete the development of intervention and trial materials, refine recruitment processes, pilot practitioner training, and confirm feasibility of intervention delivery. Over this period, the constituent parts of the SPHERe intervention were tested with four participants recruited from one centre. Full details of the development of the SPHERe intervention will be reported elsewhere.

### Internal pilot

From four NHS Trusts, 60 participants (25–30 per arm) will be enrolled to test the logistical processes of the study [21]. The pilot phase will last 6 months and will run seamlessly into the main trial if recruitment targets are achieved. As a benchmark, if recruitment is less than 50% of the target, the trial will not continue. If 50 to 75% of the target is achieved, recruitment will be reviewed at each Trust, a report will be submitted to regulatory authorities, and the trial will continue with modified recruitment strategies and close monitoring.

### Eligibility criteria

Adults with PH are eligible to participate if they meet the trial inclusion criteria **(**Table 2**):**

**Table 2:** Inclusion and exclusion criteria

Inclusion criteria	Exclusion Criteria
▪ Adults (18+) with confirmed PH (groups 1 to 5) as detailed in ESC/ERS guidelines [3]. ▪ Clinically stable: Groups 1, 4, & 5 - stable on optimal PH specific drug therapy (for those in whom it is appropriate) for at least one month, or evidence that these drugs cannot be tolerated. Groups 2 & 3 - stable on drug therapy for underlying cardiac or pulmonary disease for at least one month. Clinical stability defined as: presenting with, reproducible, manageable symptoms, not requiring any treatment other than routine follow-up care, and no PH related hospital admission in the last four weeks. ▪ World Health Organisation (WHO) functional class II, III or IV. ▪ Fluent in spoken English to allow engagement with intervention and physical outcome measures. ▪ Live within reasonable travelling distance (as defined by the participant) of a SPHERe exercise rehabilitation centre. ▪ Ability to provide informed consent.	▪ Absolute contra-indications to exercise as per international clinical guidelines [22, 23]. ▪ PH-related complications, or comorbidities severe enough to prevent attendance at a SPHERe centre, or participation in exercise rehabilitation. ▪ Any mental health issue that will prevent engagement with trial procedures. ▪ Previous randomisation in the present trial ▪ Pregnant at time of recruitment

### Participant identification, recruitment and informed consent

Participants will be identified by the clinical care team, via a number of co-ordinated screening strategies. Primarily, local secondary care disease registers and hospital discharge data (International Statistical Classification of Diseases and Related Health Problems, 10th Revision [ICD-10]) will be screened. In addition, people with less common PH aetiology (who may not be captured through other routes), and those with a high probability of PH based on echocardiographic findings [3], will be identified in specialist nurse/medical clinics.

Further to the participant information leaflet being sent in the post, and the subsequent receipt of an expression of interest form from the potential participant, a member of the clinical team at the relevant site will contact the participant and invite them to attend a baseline assessment appointment. At this face to face appointment, prior to randomisation, eligibility will be confirmed, and consent taken by an appropriately trained member of the clinical or research team. Specifically, consent will be sought for the following; 1) trial participation, 2) review of medical notes by responsible individuals, 3) collection, storage and use of personal identifiable information by authorised individuals, 4) access to long-term health and care information via NHS Digital and other NHS bodies, and 5) permission to contact next of kin.

### Randomisation, allocation concealment and blinding

Randomisation will be undertaken by Warwick Clinical Trial Unit (WCTU) centralised service using a computer-generated sequence managed by a programmer independent from the study team. Minimisation variables include centre, PH group [1–5], and WHO functional class [two categories: 1) Class II; 2) Class III or IV]. To maintain allocation concealment, all baseline data will be collected prior to randomisation. The treating practitioner will only receive the randomisation allocation electronically once all baseline measures are complete. To maintain blinding, all follow-up data will be collected by staff not directly involved in intervention delivery who are blind to treatment allocation. It is not possible to blind participants or practitioners to group allocation. Participants will be asked not to tell the assessing practitioner their group allocation when they attend their follow-up appointments. The quality of blinding will be tested by asking the outcome assessor which treatment they thought each participant had received.

### Interventions

#### The SPHERe intervention

##### Format

To ensure generalisability to the NHS, the underpinning framework of SPHERe is based on UK cardio-pulmonary rehabilitation guidelines and service delivery models [12, 24, 25], and enhanced with PH specific recommendations [3, 9, 12].

##### Cardio-pulmonary rehabilitation

Participants randomised to the SPHERe intervention will access existing cardio-pulmonary rehabilitation programmes. Service design in the UK is heterogeneous; some centres provide separate cardiac and pulmonary rehabilitation programmes, whereas others combine these programmes. This protocol will refer to these collectively as ‘cardio-pulmonary rehabilitation’.

##### Programme design

To maximise accessibility and resource, whilst ensuring that the benefits of group interaction are retained, SPHERe will be delivered as a ‘rolling’ programme. Participants randomised to the SPHERe intervention will be immediately referred to local services and invited to join existing cardio-pulmonary rehabilitation programmes running at each centre, rather than waiting for the recruitment of sufficient numbers to form a discrete group of trial participants. The SPHERe intervention has four components:

### Component 1. Individual assessment and exercise familiarisation

#### Individual assessment

A one-to-one appointment with a SPHERe ‘practitioner’ (specialist cardio-pulmonary clinical exercise physiologist or physiotherapist trained in intervention delivery). Participants will undergo an initial 1 hour ‘assessment’, as per standard practice. This will include assessment of medical history, medication, clinical parameters (i.e. height, weight, resting blood pressure, O_2_ saturation), exercise/physical activity history, and discussion of participant goals. Current exercise tolerance/capacity will be assessed with the ISWT to inform exercise prescription starting level (this is a separate test to the ISWT performed as an outcome measure).

#### Exercise prescription

The SPHERe practitioner will prescribe a tailored, individualised exercise programme within pre-specified parameters as per cardiac and pulmonary rehabilitation guidelines [12, 22, 24]. Clinical information, data from the exercise assessment, and patient centred goal setting will be used to devise a safe and effective exercise prescription.

#### Familiarisation sessions

Exercise guidance, specific to the underlying PH aetiology, will be delivered on an individual basis during two one-to-one familiarisation exercise sessions in the first week of the programme. Familiarisation sessions will allow SPHERe practitioners to refine and optimise the exercise prescription. Practitioners will begin to introduce the principles of psychosocial and motivational support during these sessions.

### Component 2. Supervised out-patient exercise programme

The programme will run within existing cardio-pulmonary rehabilitation programmes delivered by NHS clinical staff. Up to twice weekly, 1 h, supervised exercise sessions for the remaining seven to 10 weeks [12, 24] (maximum 14 sessions [16 sessions, including familiarisation sessions]), with a quantifiable and progressive dose of individualised, multi-modal, aerobic, muscular strength and endurance, and ‘functional fitness’ exercise. Adequate warm-up and cool-down will be incorporated. Intensity will be monitored and adjusted using heart rate, rating of perceived exertion, dyspnoea scale [26] and pulse oximetry (O_2_ saturation).

The SPHERe exercise component is optimised to be appropriate for a broad spectrum of patients including deconditioned, low-mobility, exercise-naive participants. It is highly adaptable to allow personalisation to lower or higher ability participants, whilst ensuring safety and efficacy. The SPHERe exercise intervention combines conventional gym-based aerobic exercise with ‘functional fitness training’. This uses multi-plane motion to target not only cardiorespiratory fitness, but also essential pre-requisites of active, independent living; e.g. agility, co-ordination, proprioception, balance and functional strength [27]. In addition to treadmills, cycle and rowing ergometers, SPHERe will make use of low-cost, readily available, functional fitness equipment; e.g. steps, floor agility ladder, low rise balance beam, power bags, plyometric boxes, ball (throw/bounce) etc. Central to SPHERe is the expertise and experience of the specialist cardio-pulmonary exercise physiologists and physiotherapist at all trial centres who will ensure holistic, safe, individualised and effective exercise training. This conforms to existing recommendations of specialist exercise supervision for this population [3, 9, 12].

### Component 3. Psychosocial and motivational support; and education

Once per week, before or after exercise, participants will receive a one-to-one 30 min psychosocial and motivational support session and a 30-min group education session (six sessions). The former will be delivered by a SPHERe practitioner, and the latter by clinical staff.

#### Psychosocial and motivational support

The aim is to improve short and long-term adherence to exercise, thus maximise benefit. As such, SPHERe will draw on social cognitive approaches to behaviour change [28], including scrutiny of multiple interactions between environment, personal factors and behaviours. Based on behaviour change techniques and the COM-B framework, three basic aspects of peoples’ lives will be addressed: capability (psychological capability through education and planning, and physical capability through supervised practice), opportunity (providing support and guidance through the different components of the programme as well as exploring external opportunities (physical and social)), and motivation (through refection and discussion of health beliefs, illness representations, monitoring of progress and exploring emotional reasoning including possible feelings of anxiety, low mood and fear) [29]. There will be a focus on increasing participants’ awareness of their priorities, through an investigation of the pros and cons of changing a specific behaviour (self-management e.g. fear avoidance of exercise) and assisting them to develop a specific plan to change (planning, goal setting). Other components of the programme will include pacing of activities, managing setbacks, stress and stress management as well as long term behaviour change. The SPHERe practitioners will be trained in motivational interviewing to assess patients’ current beliefs and encourage behaviour change. Comprehensive SPHERe manuals have been developed to guide participants and practitioners through each topic including case studies, working examples and visual aids.

#### Education

The underlying causes of PH are heterogeneous across the five PH groups [30]. It will, therefore, be essential for participants to access disease specific education. Equally, living with PH involves management of symptoms, experiences and challenges that are common to all aetiologies of PH. SPHERe participants will access both generic and disease specific group education sessions (with non-trial cardio-pulmonary rehabilitation patients), provided by clinical staff at all SPHERe centres, as part of existing cardio-pulmonary rehabilitation services. All participants (regardless of PH group) will attend generic sessions, provided as standard clinical practice by existing clinical staff, on: 1) managing breathlessness; 2) breathing control and relaxation; 3) anxiety and depression; and 4) activity pacing and energy conservation.

Disease specific topics, which may include medication, sputum clearance, managing cardiac symptoms, risk factors, smoking cessation, oxygen therapy etc., provided routinely by clinical staff, will be accessed as relevant. For people with PH groups 2 & 3, this specialist advice/education will be available as standard through the cardio-pulmonary rehabilitation programmes. Prior to enrolling in SPHERe, PH groups 1, 4, & 5 will have had discrete and extensive education with practitioners at local and national specialist clinics, as per their routine clinical treatment.

### Component 4. Guided home exercise plan

To complement supervised exercise, all participants will be provided with a manualised home exercise plan. Detailed but simple information relating to replication of the supervised exercise they have undertaken, in the home-based setting, will be provided with written instructions and diagrams in the SPHERe intervention participant manual. Each participant manual will include a diary to record time spent exercising.

### Control intervention: best practice usual care

The control arm, will be an intervention that could be described as ‘best-practice usual care’, in the form of an individual practitioner appointment, with general advice on safe and effective physical activity for those living with PH. A single 30-min appointment will allow the practitioner to discuss individualised ways in which the participant can undertake physical activity at home. Control arm participants will not be given a structured exercise plan, rather comprehensive freely available British Lung Foundation ‘Keep Active’ booklet detailing ways in which low level physical activity can be safely and effectively incorporated into everyday life. No specific psychological techniques will be used to support the provision of this information. No further intervention will be offered beyond this single advice session.

### Safety

SPHERe will be delivered in cardio-pulmonary rehabilitation units with access to emergency equipment and qualified staff. Condition-specific monitoring of exercise responses, as per cardio-pulmonary rehabilitation guidelines, will reduce and manage risk [12, 22, 24]. Guided home exercise will be lower intensity and fully manualised with instructions and photographic images. Intervention practitioners will be specialist exercise physiologists or physiotherapists, experienced in assessment, prescription and delivery of exercise in high risk clinical populations. Training in the standardised delivery of the SPHERe interventions and trial procedures will be provided for all practitioners, and bespoke manuals have been produced to guide delivery of all intervention components.

### Primary outcome

The primary outcome will be exercise capacity as determined by distance walked in the incremental shuttle walk test (ISWT) at 4 months **(**Fig. 1**)**. The ISWT will be performed as per European Respiratory Society (ERS)/American Thoracic Society (ATS) guidelines [17]. The externally paced ISWT is a simple assessment of maximal exercise capacity and, in PH, is sensitive to treatment effect, predicts mortality, and has no ceiling effect [31].

### Secondary outcomes

All outcomes will be assessed at baseline (pre-randomisation), 4 months (post randomisation) and 12 months **(**Fig. 1**)**. As a secondary measure of exercise capacity, to allow inclusion of data in future meta-analyses, the six-minute walk test (6MWT) will be performed as per guidelines [17].

Disease specific health-related quality of life (HRQoL) will be measured with the Cambridge Pulmonary Hypertension Outcome Review (CAMPHOR) [32]. This is widely used as a clinical and research tool in PH, displaying good construct validity and reproducibility. The full scale is 65 items consisting of a 25-item symptoms scale (scored 0–25), a 15-item functioning scale (scored 0–30) and a 25-item QoL scale (scored 0–25). A total score and scores for each of the three sub-scales (symptoms, functioning, QoL) are produced; for all scales, a low score indicates a better status [32].

Health utility will be assessed with the EQ-5D-5 L [33], a validated, generic HRQoL measure consisting of five dimensions, each with five levels of response. Each combination of answers can be converted into a health utility score. It has good test-retest reliability, is simple to use, and gives a single preference-based index value for health status that can be used for cost-effectiveness analysis. Anxiety and depression will be measured with the Hospital Anxiety and Depression Scale (HADS) [34], a 14-item screening questionnaire from which an anxiety and depression subscale can be derived. Sub-score values > 5 points identify increased symptoms of anxiety and/or depression; a total score > 9 is considered indicative of psychological distress. Generalised self-efficacy scale will be assessed with a 10-item psychometric scale designed to assess optimistic self-beliefs to cope with a variety of difficult demands in life; these relate to key targets of the behavioural component of the SPHERe intervention. We will capture fatigue using the Fatigue Severity Scale (FSS) [35], a nine-item questionnaire validated for evaluating disabling fatigue and previously used in PH [36]. Each item is rated on a seven-point scale, from strongly disagree to strongly agree. A total score is derived from all nine questions; a higher score indicates a greater impact of fatigue on everyday activities.

The World Health Organisation (WHO) functional class is a modified New York Heart Association functional classification system adopted by WHO and used ubiquitously in PH. Participants will be graded on their ability to perform physical tasks, and classified as (I) no limitation, (II) mild limitation, (III) marked limitation, (IV) unable to perform any activity [37]. Medication class, drug, dose and frequency of all regular medication will be recorded as well as ‘time to clinical worsening’: defined as one of; PH related death; listing for/completed lung transplant; hospitalisation for PH; clinical worsening leading to initiation of new PH treatment; decreased WHO functional class and ≥ 15% decrease in 6MWT distance [38]. Time to clinical worsening will be measured as the time that has elapsed since randomisation to the SPHERe trial.

Health and social care resource use will be evaluated with participant self-report and NHS records. The primary health-economic analysis will concentrate on direct intervention and healthcare/personal social services costs, while wider impact (societal) costs will be included within the sensitivity analyses. Participants will complete resource use questionnaires in person or by post at four and 12 months, to collect resource use data associated with the interventions. Participants will be provided with a resource use diary as an aide memoire to help record resource use between four and 12 months. At the end of the follow-up period, a copy of medical records for the participant will be requested from their GP. This will provide information on GP consultations and include copies of any hospital discharge letters allowing accurate costing of in-patient care costs. Where appropriate, data will be triangulated from GP records, participant self-report, and data held in participating hospitals, to achieve a robust estimate of health service activity.

Finally, all-cause hospital admissions will be identified from GP records, adverse events recorded as per good clinical practice (GCP) guidelines, and all-cause mortality flagged via NHS digital to ensure notification of any deaths and cause of death both during the trial and for longer term follow-up.

### Follow-up

Outcomes will be assessed at 4 months and 12 months post randomisation **(**Fig. 1**)**. The primary outcome is an objective measure of exercise capacity for which participants will attend their treatment centre. Patient reported outcomes will also be collected at follow-up assessments. If any participants are unable to attend clinic, a postal questionnaire will be used to collect patient reported outcomes. In the case of non-response, two key secondary outcomes (CAMPHOR and EQ-5D-5 L) will be collected by phone. For long-term follow-up, consent will be sought from participants to keep their personal data, and have access to their NHS data following the end of the current trial. This will allow longer term postal follow up to assess QoL and to monitor deaths using NHS Digital data.

### Sample size

The primary outcome will be distance walked measured using the ISWT at 4 months post-randomisation. As there are no directly applicable ISWT data with which to calculate a sample size, or previously defined worthwhile effect sizes for IWST in people with PH, 6MWT data have been used to estimate the sample size. The 6MWT distance, unlike the alternative approach of using a standardised mean difference (SMD), has the advantage that it is meaningful to participants and grounded in clinical reality.

The baseline pooled 6MWT distance in current studies of exercise rehabilitation for PH is 414 m (SD 91) [16]. Whilst a useful starting point, these data indicate a comparatively fit group of people with PH (younger, group 1 PH). Based on a sample from one cardio-pulmonary rehabilitation service at UHCW NHS Trust, typically, people with PH walk around 300 m in the 6MWT. Conventionally, the minimally clinically important difference for PH studies is 30 m on 6MWT or an SMD of 0.33 [17]. Patient partners suggested that a larger difference was needed to make this treatment worthwhile. Therefore, sample size is predicated on showing a mean difference of 45 m in 6MWT distance. This equates to a standardised mean difference of 0.5; conventionally a moderate effect size.

To achieve 90% power at 5% significance level, to show a difference in 6MWT distance of 45 m, with a standard deviation of 90, data from 170 people are needed. Allowance has been made for clustering effects by site in the intervention arm using Moerbeek’s method [39] and an unbalanced randomisation. Therefore, an unequal allocation (1.15:1 for intervention vs control) was determined based on the following assumptions: a mean cluster size of 12 at follow-up, an intra-class correlation co-efficient (ICC) of 0.03 and same group variance. The ICC is an overall estimation of the site and practitioner effect, leading to an estimated design effect of 1.33, although a negligible practitioner effect in this trial is anticipated. Accordingly, the group sample sizes were calculated separately, given the power of 90% and a significance level of 5%. An 80% retention rate is expected at 4 months. Therefore, 246 participants (132 in intervention) will be recruited to allow for 20% loss to follow-up.

The primary aim is to show an overall effect size for all PH groups without considering participant mix. Based on published data for prevalence of PAH and CTEPH, however, most participants will have PH groups 2 or 3. Sufficient data will be collected to assess outcome in a pooled group of people with group 2 or 3 PH as a secondary analysis. Approximately 70% of the total sample size will be people with group 2 or 3 PH. To ensure power of 90% power, for this sub-group, the sample of 246 will inflated to 352 participants (189 intervention, 163 control). This will be the total sample for the trial which will ensure sufficient power for the main analysis as well as the sub-group analysis. There is some uncertainty about the final sample size because of the need to include 246 people with group 2/3 PH in the overall population and an ambition to include a minimum of 20 people each from PH groups 1, 4, & 5.

### Data analysis

The main analyses will be for overall treatment effect regardless of PH diagnostic group. Data will be summarised and reported in accordance with CONSORT guidelines for RCTs, using intention-to-treat analyses [40]. Hierarchical linear regression models will be used to estimate the treatment effects (95% confidence intervals), adjusted for important patient-level covariates and centre effect. Estimation of, and adjustment for practitioner effects will be included. If there is negligible practitioner and centre effect, then the usual linear regression will be used for the analysis. Categorical data will be assessed in a similar way, using logistic regression models. The main analyses will be intention to treat and will assess the impact of compliance on outcomes using a CACE (complier average causal effect) analysis. For the intervention group, full compliance will be considered as attending at least 75% of the supervised exercise sessions. In addition, probabilities for achieving the desired effect size in each of the PH groups will be presented using the magnitude based inference approach [41]..

In a planned secondary analysis the pooled effects for PH groups 2 & 3 will be presented. Pre-specified sub-group analyses will examine the interaction of treatment assignment with the groupings of PH. Analysis will be conducted using formal tests of interaction. This trial is not powered to identify interactions, thus, whilst pre-specified, these analyses should be considered as no more than exploratory. However, the effect size for pooled PH groups 2 & 3 will be presented as a separate analysis. Main outcomes will also be presented by diagnostic group (minimum 20 people contributing data) to inform decision makers and guidance developers interested in specific PH groups. To maximise data value, data from published trials (identified in an updated systematic review) assessing the same outcomes in RCTs of out-patient/community outreach interventions for specific PH group, will be included. The statistical methods will be further elaborated in a statistical analysis plan (SAP).

### Health economic evaluation and analysis

A prospective economic evaluation, informed by the NICE Reference Case [42], will be finalised within a Health Economics Analysis plan (HEAP), prior to any analysis. The primary perspective will include NHS and Personal Social Services (PSS) costs. However, patient direct and indirect costs will also be included in a secondary broader societal perspective. Primary care and referral events will be captured both from health records and from participants, using a triangulation and adjudication approach to promote robust estimates of resource use. Participants will also report PSS and personal direct and indirect costs. Personal Social Services Research Unit (PSSRU) [43] and national hospital reference costs [44] will be used as principal unit cost sources. Patient level costs will be estimated by combining resource use data with unit cost. Intervention costing will reflect the structure within which care is being given and will, by necessity, balance precision with practicality.

EQ-5D-5 L responses will be used to generate quality-adjusted life years (QALYs) using the UK value set recommended by NICE guidance at time of analysis [45]. These health state values will be used to estimate QALYs at the patient level, over 1 year, using the trapezoidal rule. The EQ-5D-5 L will be used as the overall HRQoL outcome due to specific concerns about the sensitivity to change of other measures such as the SF-36, in this population. The EQ-5D-5 L is likely to be more responsive to change than the 3 L, and hence is preferred as a clinical outcome. Significant adverse events will be captured summatively in the HRQoL estimation. Decision modelling will be considered beyond the end of the trial if outcomes have not converged.

It is anticipated that bivariate regression of costs and QALYs (with bootstrapping of models) will be used to generate incremental cost per QALY estimates and credible intervals, cost-effectiveness acceptability curves, and value-of-information analysis. With regard to normality, invoking the central limit theorem avoids the problems that non-Gaussian link functions generate for the analysis. However, if distributions are very unusual, cost and QALYs will be conflated in a net benefit analysis evaluated at different thresholds of willingness to pay, reducing the analysis to a univariate regression problem.

### Embedded process evaluation

Semi-structured interviews will be conducted in person or on the phone/video call as appropriate. Intervention and control participants will be interviewed to investigate their experiences, contextualise quantitative findings, and explore factors that helped or hindered participation, thus informing interpretation and wider implementation. Interviews will take place after the 12-month follow-up outcome data collection, so that the interview itself does not introduce bias to the analysis. A purposive sample of up to *n* = 20 intervention, *n* = 20 control and *n* = 20 practitioner will be interviewed to ensure a diverse range of perspectives are included. The interviews will use a topic guide that will include participant response to the intervention (or control), what they found difficult, what worked well, specific obstacles and enablers, what components were used/dropped/never used, and views on the guided home exercise content. Interviews will last about 1 h, be digitally recorded, pseudo-anonymised, and transcribed verbatim. Data will be analysed using the Framework method [46]. Quantitative and qualitative data will be integrated using a mixed methods matrix’ where quantitative responses can be compared to interview data [47] as described elsewhere in the SPHERe intervention development publication.

All psychosocial/motivational sessions and control (1:1 session) sessions will be recorded. A purposively selected subset (10%) of recordings will be analysed, with a checklist to assess fidelity and help understand which areas generated discussion. Intervention fidelity will be assessed using the tenets highlighted by Mars et al. [47].

### Data management and security

All essential documentation and trial records will be stored in conformance with the applicable regulatory requirements, and access restricted to authorised personnel. Electronic data will be stored on password protected computers in a restricted access building. All data will be pseudonymised after the collection of the baseline demographic data. For quality assurance, the data and results will be routinely checked for completeness and accuracy. Trial documentation and data will be archived for 10 years after completion of the trial.

### Trial management and monitoring

The Trial Management Group, consisting of project staff and co-investigators involved in the day-to-day running of the trial, will meet monthly throughout the project. Any significant issues will be referred to the Trial Steering Committee (TSC) which will meet twice yearly and constitute a group of experienced personnel and trialists, ‘lay’ representatives, and an independent Chairperson. A Data Monitoring Committee (DMC) consisting of independent experts with relevant clinical research and statistical experience, will meet twice yearly to ensure data integrity and participant safety.

### Adverse event management

An Adverse Event (AE) will be defined as any untoward medical occurrence in a participant which does not necessarily have a causal relationship with the intervention. Any AEs related to SPHERe will be recorded and reported to the relevant oversight committees. In this population, serious adverse events (SAE) are expected. Hospital admissions data will be collected through self-report and GP records, and deaths via NHS Digital. SAEs that have no causal relationship with the intervention will not be reported to oversight committees. Causality and expectedness will be confirmed by the CI with clinician support. SAEs deemed to be unexpected and possibly, probably or definitely related to the trial interventions will be notified to the Research Ethics Committee (REC) within 15 days. All AEs and SAEs will be recorded within 24 h of the investigator being made aware.

### Patient and public involvement

The SPHERe intervention and trial were developed during the grant funding application process and subsequently during the pre-pilot feasibility phase. Patient and public involvement was pivotal at every stage. A three-stage process was followed as per MRC guidance: 1) systematic literature review; 2) expert opinion, stakeholder engagement and consensus meetings; 3) intervention piloting, acceptability and refinement. Lay partners and co-applicants were fully integrated into every stage of trial development, taking an active role in refining intervention components and reviewing the application. A lay co-applicant currently sits on the trial management group (TMG), initially meeting monthly and subsequently quarterly, and has a pivotal role in steering the conduct of the trial. She reviewed the ethics application to ensure that trial documentation e.g. participant information leaflet, was user appropriate. Lay partners have been actively involved in trial publicity and media and will help with the dissemination of findings through appropriate channels i.e. social media, lay conferences, public engagement events, service provider events, newsletter articles. Lay partners also sit on the TSC as verified independent members. Lay co-apps and partners are supported by the chief investigator (CI), trial management team, and through the peer support of lay partners on existing clinical trials. Comprehensive training and support was provided by WCTU.

## Discussion

Pulmonary hypertension is life-limiting and can have a substantial impact on QoL [2]. Amongst numerous debilitating symptoms, exertional breathlessness and fatigue prevent completion of many activities of daily living [1]. For PH groups 2&3 in particular, there is a distinct lack of targeted therapies and treatment options.

Exercise rehabilitation is routinely offered to people with many forms of heart and lung disease, but due to a lack of empirical data, is not currently provided for people with PH. The SPHERe study aims to address this evidence gap. It is the first multi-centre clinical trial to assess the clinical and cost effectiveness of a supervised out-patient exercise rehabilitation intervention compared to best-practice usual care, delivered in the UK NHS, for people in all PH diagnostic groups.

Results from the SPHERe study will inform many areas of clinical practice. Firstly, clinicians and rehabilitation practitioners will gain invaluable insight into the specific exercise rehabilitation requirements of people with PH. Secondly, they will have definitive answers as to the clinical efficacy of NHS out-patient exercise rehabilitation programmes for this diverse population. Thirdly, commissioners will be able to appraise the cost-effectiveness of such programmes and inform commissioning strategies accordingly. Finally, people with all forms of PH will be able to get an appreciation for the potential benefit or harm of out-patient exercise rehabilitation and make informed decisions as to their future participation in physical activity and exercise programmes. Should the trial find that exercise rehabilitation is beneficial to health-related outcomes, the SPHERe supervised and home-based exercise rehabilitation resources will be made freely available to rehabilitation practitioners and people with PH.

### Trial status

Recruitment to the internal pilot began in January 2020 and was subsequently temporarily suspended in March 2020 due to the Covid-19 pandemic. Trial activities are expected to recommence later in the year.

### Supplementary information

**Additional file 1.** SPIRIT 2013 Checklist: Recommended items to address in a clinical trial protocol and related documents*.

## Data Availability

The datasets used and/or analysed during the current study will be available from the corresponding author on reasonable request.

## Abbreviations

AE: Adverse Event
CACE: Complier average causal effect
CAMPHOR: Cambridge Pulmonary Hypertension Outcome Review
CHF: Chronic heart failure
CI: Chief investigator
CONSORT: Consolidated Standards of Reporting Trials
COPD: Chronic obstructive pulmonary disease
CTEPH: Chronic thromboembolic pulmonary hypertension
DMC: Data Monitoring Committee
FSS: Fatigue severity scale
GCP: Good Clinical Practice
HADS: Hospital anxiety and depression scale
HEAP: Health economics analysis plan
ICC: Intra-class correlation co-efficient
IRAS: Integrated Research Application System
ISRCTN: International Standard Randomised Controlled Trial Number
ISWT: Incremental shuttle walk test
NICE: National Institute for Health and Care Excellence
MRC: Medical Research Council
PAH: Pulmonary arterial hypertension
PH: Pulmonary hypertension
PI: Principle investigator
PPI: Patient & public involvement
PSS: Personal social services
PSSRU: Personal social services research unit
QALYs: Quality-adjusted life years
HRQoL: Health-related quality of life
RCT: Randomised controlled trial
REC: Research Ethics Committee
R&D: Research and development
SAE: Serious adverse event
SAP: Statistical analysis plan.
SOP: Standard operating procedure
SMD: Standardised mean difference
TSC: Trial steering committee
UHCW: University Hospitals Coventry & Warwickshire
WCTU: Warwick Clinical Trials Unit
WHO: World Health Organisation
6MWT: Six-minute walk test
